# Riedel's Procedure: A Modification to Obliterate Step Defect

**DOI:** 10.1155/2019/9437641

**Published:** 2019-02-17

**Authors:** Abdul Nassimizadeh, Mohammad Nassimizadeh, Shahzada Ahmed

**Affiliations:** Queen Elizabeth Hospital, Birmingham, Mindelsholn Way, Edgbaston, Birmingham B15 2WB, UK

## Abstract

Sinus surgery was first introduced by the ancient Egyptians. In 1750 was the first modern description of frontal sinus surgery. In 1898, Riedel advocated complete removal of the anterior table and floor of the frontal sinus while simultaneously stripping the mucosa. The major postoperative issue involved gross forehead deformity. We aim to provide a modification to reduce the postoperative “step” defect. Riedel's procedure is an effective way of managing frontal sinus disease when endoscopic surgery has repetitively failed. Use of a pedicled pericranial/galeal soft tissue flap can effectively reduce cosmetic deformity postoperatively.

## 1. Introduction

Although sinus surgery was first introduced by the ancient Egyptians, it was not until 1750 where we encountered the first modern description of frontal sinus surgery. This began the era of trephination, characterised by the Ogston–Luc procedure, which involved dilatation of the nasofrontal duct. Despite initial success, this method of frontal sinus management often failed due to duct stenosis. In 1898, Riedel advocated complete removal of the anterior table and floor of the frontal sinus, while simultaneously stripping the mucosa in a patient with osteomyelitis. The posterior wall was retained to separate intracranial contents. The major postoperative issue involved gross forehead deformity [1, 2]. Killian attempted to modify the procedure by retaining a 1 cm bar of supraorbital rim but faced multiple complications including stenosis, supraorbital rim necrosis, postoperative meningitis, and mucocoele formation [1]. The 20th century heralded the start of conservative management modified to current day endoscopic practice.

Even with over a century of advancement, Riedel's procedure and other forms of osteoplastic frontal surgery still play pivotal roles in a small select proportion of patients with frontal sinus disease [2–4]. These include patients with osteomyelitis of the anterior wall of the frontal sinus, chronic refractory frontal osteomyelitis that has defied conventional management, failure of frontal sinus obliteration, frontal sinus access when AP diameter is less than 5 mm, and frontal sinus tumours unsuitable for endoscopic surgical management. The biggest criticism continues to be postoperative disfigurement. Previous modifications included reconstruction at later dates and extensive chamfering of the margins [5]. We present a patient undergoing Riedel's procedure and important technique modifications to improve postoperative cosmesis.

## 2. Patient Presentation

**Figure 1:**
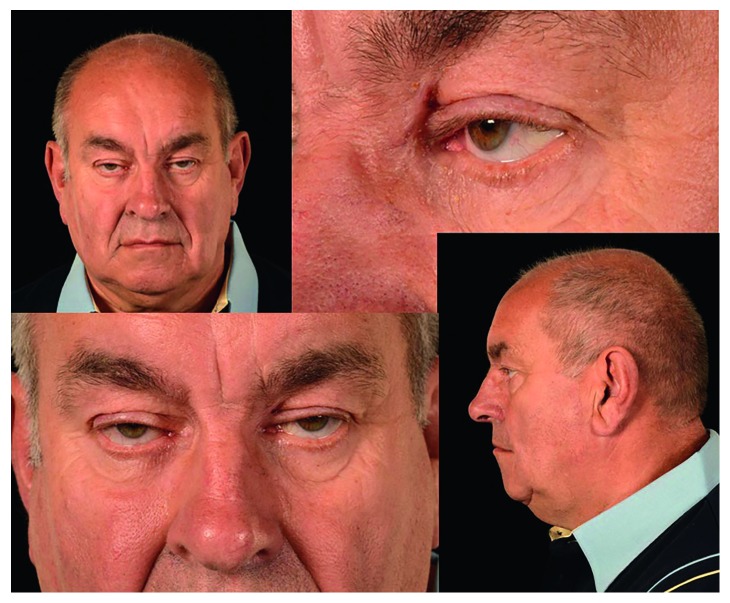
Preoperative images. Left fronto-orbital fistula draining into the supra-medial orbit.

A 74-year-old male (MF) was referred to our tertiary centre following two previous endoscopic endonasal sinus operations during the preceding 18 months prior to presentation. This was performed for chronic rhinosinusitis and left frontal mucocoele. His medical history also included significant ptosis, despite previous ophthalmological intervention. At presentation, he suffered from recurrent sinonasal disease with left-sided headache and left fronto-orbital fistula discharge over his medial canthus (Figure 1).

**Figure 2:**
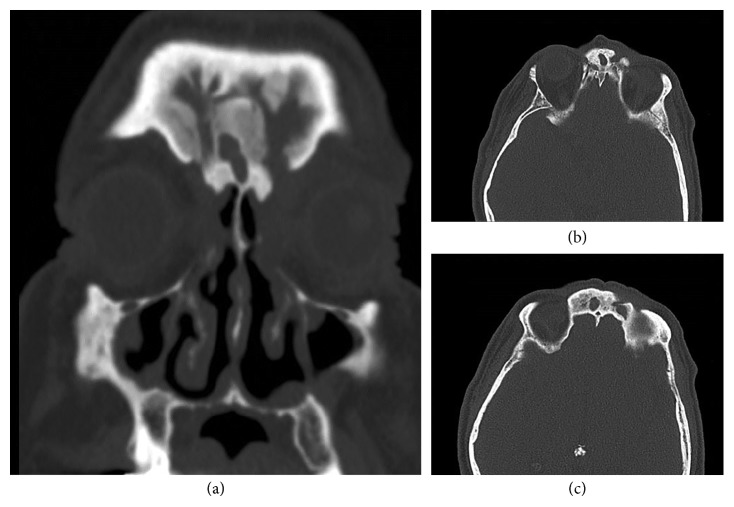
CT orbit, sinuses, and skull base. Small A-P diameter of frontal sinus with extensive neo-osteogenesis secondary to chronic frontal sinusitis.

MF underwent computer tomography of the orbit, sinuses, and skull base (Figure 2), demonstrating the small A-P diameter of frontal sinus and extensive neo-osteogenesis from chronic frontal sinusitis. An endonasal endoscopic approach would likely be ineffective in this patient. Multidisciplinary discussion led to the option of Riedel's procedure and concurrent excision of fronto-orbital fistula under the same anaesthetic.

## 3. Operative Methodology

**Figure 3:**
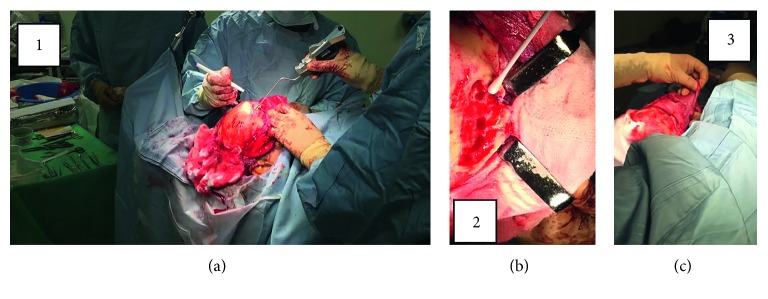
Operative methodology. (a) Bicoronal approach under image guidance used to mark out frontal sinus and lateral limits. (b) Washout and debridement of infective mucosa and bone (subsequent extensive chamfering). (c) Raising of thick pericranium tissue which included periosteum, galea muscle, and some temporalis.

**Figure 4:**
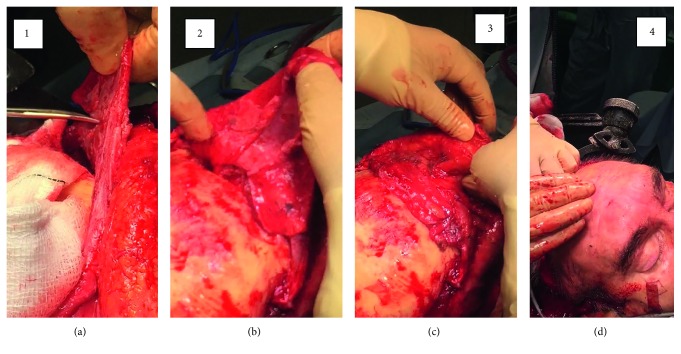
Obliteration of sinus defect. (a) Incisions bilaterally to improve manipulation capacity. (b) Roll edges of pedicled flap into sinus defect. (c) Central compartment folded over to smoothen sinus defect. (d) Replace skin, with further external manipulation of pericranial/galeal soft tissue flap.

MF underwent bicoronal approach using image guidance to mark the lateral limits of the frontal sinus (Figures 3 and 4). The anterior wall of frontal sinus was removed. Following this, there was thorough washout and debridement of infective mucosa and bone, in addition to extensive chamfering of the edges. A typical Riedel's procedure would finish following replacement of the bicoronal skin flap.

Modification involved raising thick vascularised pericranium tissue including periosteam, galea muscle, and some temporalis muscle. Accurate placement of the pedicled soft tissue flap involved folding to create bulk, before tightly placing the flap within the sinus cavity. This was effective in completely obliterating the sinus defect with minimal cosmetic deformity (Figure 4).

**Figure 5:**
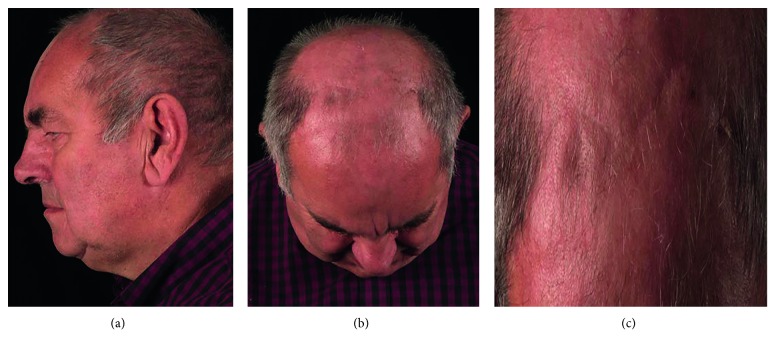
Postoperative images (12 months).

**Figure 6:**
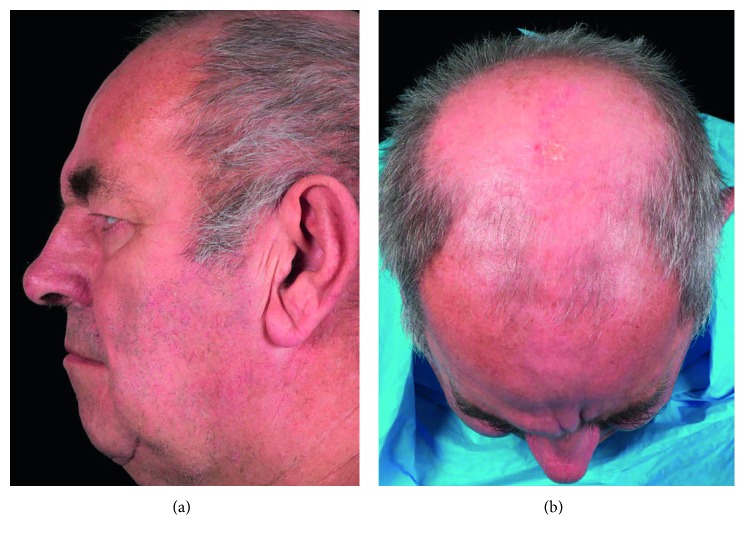
Postoperative images (24 months).

Postoperatively (Figures 5 and 6), the patient had excellent cosmetic results immediately, as well at 12 and 24 months follow-up. No obvious step deformity can be seen with healed bicoronal scar despite male pattern balding.

## 4. Discussion

Most surgical interventions are not on public display. As a result, they cause less stigma than head and neck surgery. Within the world of sinus surgery, the frontal sinus involves the greatest challenge for management. Challenges consist of anatomical considerations including diploe of the anterior and posterior table inviting osteomyelitis, diploic veins being potential sources of intracranial infections, and a long, irregular outflow tract which can possibly be impinged by extramural ethmoid cells. Physiological considerations such as mucociliary flow into the nasal cavity introducing a further source of infection must similarly be addressed. It is also the site with the greatest source of mucocoeles. Due to these difficulties encountered, the frontal sinus continues to be a difficult site to manage despite technological advances [6].

A majority of patients in previous studies undergoing sinus obliteration were secondary to chronic refractory infection. Most infective pathology involving the frontal sinus is managed effectively using endoscopic or external drainage alongside a prolonged course of intravenous antibiotics. In a subset though, infection can persist. Contributing factors include nasofrontal patency, persistent infected bone, and secondary mucocoele formation. Other factors reported as indications for sinus obliteration include trauma, subcutaneous fistula, periorbital collections, and epidural and subdural abscesses [6–8].

With regard to sinus obliteration, over the years, there have been multiple attempts to improve postoperative disfigurement. These have included the use of autogenous fat, auto-obliteration by osteoneogenesis, and cranialization by removing the posterior table which allows herniation of the brain and dura against the anterior table. More recent treatment involves the use of alloplastic material or autogenous bone graft [1, 6–8].

Current literature is limited with regard to sinus obliteration. Patients requiring obliterative frontal sinus procedures vary between two and five in most papers. These are often across multiple decades of work. Long-term complications mentioned include cerebrospinal fluid leak, encephalocoele, and intracranial and epidural abscesses [1, 6–8].

The shortcomings of our procedure include the limited length of time of follow-up (currently 28 months). Despite this, we have not encountered any postoperative complications. There is however no definitive way of ascertaining the long-term sequelae and postoperative changes.

We advocate the use of pedicled soft tissue flap. With proven links between facial profile and identity, distortions can lead to reduced self-confidence, psychological and adjustment issues, and overall increase in postoperative morbidity. This remains a possibility despite minimal body image distortions postoperatively, ultimately leading to psychological problems inhibiting the positive effects of surgery [9]. As a result, despite only minimal numbers of patients worldwide requiring sinus obliteration, improvement of a surgical technique first reported in the late 19th century is imperative. We present a surgical technique modification which addresses postoperative disfigurement significantly.

## 5. Conclusion

Riedel's procedure is no longer commonly used with the advancement of endoscopic endonasal approaches. However, there are rare instances this procedure would be useful. In those circumstances, we believe a vascularised pedicled flap would be useful to improve postoperative morbidity and appearance.

## Additional Points

(1) Riedel's procedure is an effective way of managing frontal sinus disease when endoscopic surgery has repetitively failed. (2) It is typically associated with a postoperative step defect. (3) Use of a pedicled pericranial/galeal soft tissue flap can effectively reduce cosmetic deformity postoperatively.
